# VGG16 Feature Extractor with Extreme Gradient Boost Classifier for Pancreas Cancer Prediction

**DOI:** 10.3390/jimaging9070138

**Published:** 2023-07-07

**Authors:** Wilson Bakasa, Serestina Viriri

**Affiliations:** School of Mathematics, Statistics and Computer Science, University of KwaZulu-Natal, Durban 4041, South Africa; 219098448@stu.ukzn.ac.za

**Keywords:** feature extraction, classification, computerised tomography, VGG16, XGBoost

## Abstract

The prognosis of patients with pancreatic ductal adenocarcinoma (PDAC) is greatly improved by an early and accurate diagnosis. Several studies have created automated methods to forecast PDAC development utilising various medical imaging modalities. These papers give a general overview of the classification, segmentation, or grading of many cancer types utilising conventional machine learning techniques and hand-engineered characteristics, including pancreatic cancer. This study uses cutting-edge deep learning techniques to identify PDAC utilising computerised tomography (CT) medical imaging modalities. This work suggests that the hybrid model VGG16–XGBoost (VGG16—backbone feature extractor and Extreme Gradient Boosting—classifier) for PDAC images. According to studies, the proposed hybrid model performs better, obtaining an accuracy of 0.97 and a weighted F1 score of 0.97 for the dataset under study. The experimental validation of the VGG16–XGBoost model uses the Cancer Imaging Archive (TCIA) public access dataset, which has pancreas CT images. The results of this study can be extremely helpful for PDAC diagnosis from computerised tomography (CT) pancreas images, categorising them into five different tumours (T), node (N), and metastases (M) (TNM) staging system class labels, which are T0, T1, T2, T3, and T4.

## 1. Introduction

Machine learning uses enormous datasets that require a lot of processing power to produce predictions and recommendations that are more accurate. The efficient feature extraction (FE) [1] technique allows for resource reduction without sacrificing important data. Machine learning models can be made more effective and accurate by extracting features. The amount of redundant data in the collection is decreased with feature extraction. In the end, the data reduction speeds up the learning and generalisation phases of the machine learning process while also enabling the model to be built with less machine effort [2]. Extracting the features can be helpful when one has a large dataset and needs to conserve resources without sacrificing any crucial or pertinent data. Finally, the data reduction speeds up the learning and generalisation phases of the machine learning process while requiring less machine work to develop the model.

Machine learning is more accurate and effective when features are extracted. By removing redundant and unwanted data, feature extraction cuts through the noise. Only the data necessary to train the model for its intended medical diagnosis use are necessary to build the most accurate machine learning models [3]. The model’s accuracy suffers with the inclusion of ancillary data. The learning process is slowed by using training data that are not specifically relevant. A reduction in data means processing jobs that do not bring value do not occupy computational resources.

In image analysis activities, image classification poses a significant problem, particularly regarding the choice of methodologies and strategies for utilising the output of image processing and pattern recognition, classification methods, and, ultimately, verifying the image classification result against medical expert knowledge [4]. In addition to achieving high accuracy, the primary goal of medical image classification is to pinpoint the specific areas of the pancreas that are infected.

However, extracting the right image features to capture the essential information from a dataset accurately is still difficult. This study suggests using a hybrid VGG16–Extreme Gradient Boost (XGBoost) model to extract and classify pancreatic ductal adenocarcinoma (PDAC) features from computerised tomography (CT) images. There are stages 0 (zero) and I to IV (1 to 4). For clinicians to collaborate and develop the most effective treatment plans, the use of stages offers a common manner of classifying cancer. The “T” plus a letter or number (0 to 4) in the tumour (T), node (N), and metastases (M) (TNM) staging system [5,6] is used to indicate the size and location of the tumour. Centimetres (cm) are used to assess tumour size. A normal pen or pencil’s width is about equivalent to one centimetre. The tumour stage aids the physician in creating the most effective treatment strategy for each patient. Details on each tumour stage are listed below:

- T0 (time plus 0): There are no signs of cancer in the pancreas;
- T1: The tumour is solely in the pancreas and is no more than 2 cm in size (according to item T1). T1a, T1b, and T1c are other stages that can be identified based on the size of the tumour;
- T2: The tumour, which is only in the pancreas, is more than 2 cm but less than 4 cm in size;
- T3: The tumour is larger than 4 cm and extends past the pancreas. There is no involvement of the major arteries or veins near the pancreas;
- T4: The tumour spread affects the pancreas and nearby major arteries and veins. A T4 tumour cannot be completely removed during surgery.

Each PDAC tumour must be separately diagnosed because it may affect the prognosis and course of treatment. Accurate identification of every PDAC multi-classification category is necessary for proper diagnosis.

Deep learning [7,8,9] allows computers to understand images and learn from facts. This intelligence allows computers to extract patterns which are particular to a dataset and use those patterns to support autonomous reasoning. Based on some expectations, digital image processing considerably influences decision-making processes. It provides more accurate feature extraction.

1. XGBoost classifier [10], pre-trained VGG16 model [11,12], and deep learning were combined to help with the early diagnosis of PDAC patients;
2. Nine different combinations, including VGG16–XGBoost, VGG16–RF, and VGG16–SVM, Inception V3–XGBoost, Inception V3–RF, and Inception V3–SVM, as well as LGBM–XGBoost, LGBM–RF, and LGBM–SVM, were tested to identify PDAC in pancreas CT images. In these combinations, XGBoost, RF [13,14], and SVM [15] were employed as classifiers, while Inception V3, VGG16, and LGBM [16] were used as deep feature extractors;
3. To evaluate the effectiveness of the proposed framework in terms of accuracy, precision, recall, F1-score, and confusion matrix, a detailed experimental investigation was performed.

**Figure 1:**
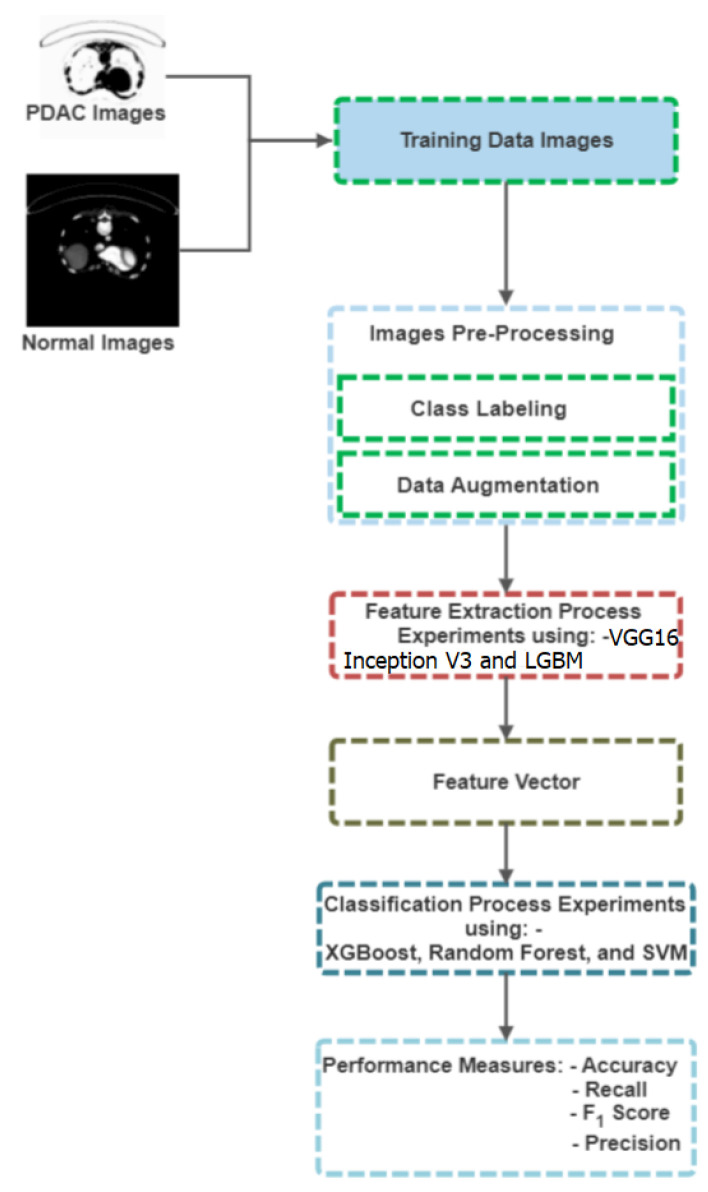
Different steps to follow when using deep learning algorithms in medical image processing.

Several deep learning algorithm phases are applied in medical imaging, as illustrated in Figure 1.

Developing algorithms and models to assist radiologists in detecting and diagnosing diseases and applying machine learning techniques to improve image processing and diagnosis are among the difficulties confronting computer-aided diagnosis [17]. Another problem is improving image visibility or diagnostic quality so that discrete structures or regions of interest within an image can be detected and separated. Image registration, or aligning images received in different modalities or at different times to make them more comparable, should also be considered.

One of the most significant issues in image recognition is classifying medical images into several groups to aid in diagnosing diseases or further scientific investigation [18]. Two steps can be used to classify medical images in general. Finding useful elements in the image is the first step. In the following step, classification models for the dataset of images are created utilising the features. The classification of medical images into several classes has traditionally been a challenging, tedious, and time-consuming task performed by doctors using their professional experience. This strategy is likely to provide unstable or unpredictable results. Medical image classification application study has had significant worth compared to prior research [19]. Numerous papers in this field have been published due to the scholars’ work. However, as of right now, we are still unable to complete our objective efficiently. Doctors could diagnose ailments with additional research if we complete the categorisation task. Consequently, figuring out how to complete this activity effectively is crucial.

The main objectives of this study are to automate the diagnosis and to categorise phases of PDAC from CT images with the same accuracy as that of an expert in medical science. This paper contributes to building a hybrid classification model by taking advantage of different algorithms used in deep learning to address the above problems. VGG16 is used to extract features from images and classify them using XGBoost. To do this, we propose the VGG16–XGBoost model.

The automatic diagnosis of diseases on CT images is primarily improved by a good classification model [20]. This is necessary so that the diagnostic algorithms can adjust to the image groupings produced due to the categorisation. Firstly, this study aims to automatically recognise and classify medical images using deep learning (DL) algorithms—VGG16, Inception V3, and LGBM—to aid in the diagnosis, treatment planning, and monitoring of illness. Secondly, this study experiments and compares three model classifiers: XGBoost, Support Vector Machines (SVM) and Random Forest (RF). Lastly, after experimenting on individual models, the study aims to use a hybrid model to extract and classify PDAC features from CT images [21].

Our literature analysis shows that, before developing deep architectures, many researchers have classified medical images using shallow models, which primarily rely on shape, colour, texture elements, and their combinations [22]. The biggest issue with these models is that the extracted features are sometimes referred to as low-level features; these features have weak generalisation abilities and cannot represent high-level concepts in the problem domain. On the other hand, the non-medical imaging area has seen a lot of success with deep architecture. Deep learning-based methods, the most amazing branch of machine learning, are an efficient technique to build an end-to-end model that can calculate final classification labels using the raw pixels of medical images.

## 2. Related Work

Ref. [23] suggested machine learning algorithms that aid in diabetes prediction and found that LGBM performed better than all other models, with an accuracy of 89.85% and an area under the ROC curve (AUC) of 0.95. As a result, LGBM is a more effective algorithm for differentiating between diabetes and non-diabetic individuals.

Using a few selected features, [24] classified colon cancer tissues using SVM. They used 40 colon cancer tumours and 22 healthy colon tissues and obtained an accuracy of 94.51%. The top 50–200 genes were then utilised for training SVMs, which distinguished between non–tumour and tumour specimens as well as or better than the entire repertoire of 1988 genes.

On the patient dataset for COVID-19, [25] offered a model to use the RF approach, which had an F1 Score of 0.866 and was enhanced by the AdaBoost algorithm. On unbalanced datasets, the Boosted Random Forest algorithm also provided detailed forecasts and an accuracy of 94%. The information analysed in this investigation showed that Wuhan natives had higher death rates.

To classify brain tumours from MRI scans, [26] used both VGG16 and AlexNet, with each model capturing 1000 characteristics. The collected features were further assessed using the recursive feature elimination (RFE) selection technique to determine the most effective features. With 200 features selected, the SVM classifier achieved 96.77% accuracy. An SVM for pneumonia identification using X-rays was presented by Eid and Elawady, based on ResNet. The created model employed an SVM classifier to identify pneumonia based on the relevant features after using a boosting technique to choose the features from chest X-rays that were important to the problem [27]. Following training with 5863 X-rays, the model’s accuracy was 98.13%.

## 3. Materials and Methods

The feature extraction process converts unprocessed data into numerical features that machine learning algorithms can use. Data scientists can develop new features appropriate for machine learning applications by extracting the geometry of an object or the redness value from images. A crucial aspect of image processing is feature extraction [28]. This method is used with other devices to identify elements in digital images, such as edges, forms, or movements. Once these have been located, the data can be processed to carry out various functions relating to image analysis.

Utilising feature descriptors such as Scale-Invariant Feature Transform (SIFT) and speeded-up robust features (SURF) as a method for object detection, using well-established Computer Vision (CV) algorithms is the conventional method [29]. For applications such as image classification before the development of DL, a procedure called feature extraction was used. Features are discrete areas of an image that are “interesting”, “descriptive”, or “informative”. This stage might incorporate CV methods, including threshold segmentation, corner detection, and edge detection. A bag-of-words, or definition, of each object class is created using the most features that can be practically derived from an image [30]. During the deployment phase, these definitions are looked up in other images. An image is labelled as containing a particular object if it contains a sizeable number of features from one bag of words in another image.

**Figure 2:**
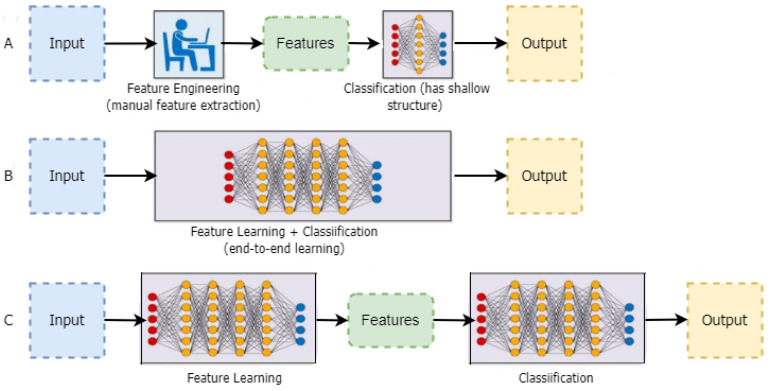
(**A**) The classification process using traditional techniques, (**B**) the classification process using DL techniques, and (**C**) the classification process using the proposed model.

End-to-end learning is a concept introduced by deep learning (DL) [31]. In this approach, the computer is given a dataset of images tagged with the classes of objects present in each image. As a result, a DL model is “trained” on the input data, where neural networks identify the underlying patterns in class images and automatically determine which features are the most salient and descriptive for each object in each class [32]. Although there are trade-offs in computing resources and training time, it is widely documented that DNNs outperform conventional techniques. The CV engineer’s workflow has significantly changed due to all state-of-the-art approaches in CV using this methodology. Instead of extracting hand-crafted features, which was once their area of expertise, they now iterate through deep learning architectures, as shown in Figure 2.

Three algorithms, VGG16, LGBM, and Inception V3, were experimented on to find which one would extract highly accurate features and consider different metrics. Three classifiers, SVM, RF, and XGBoost, were also experimented on to find the one which gave high accuracy. The best feature extraction, VGG16, was combined with the best classifier, XGBoost, to solve the problem and obtain better outcomes. First, experiments compared three deep learning (DL) feature descriptors: VGG16, Inception V3 and LGBM. Secondly, experiments compared three model classifiers: XGBoost, Support Vector Machines (SVM), and Random Forest (RF). Lastly, after experimenting on individual models, the study used a hybrid model to extract and classify PDAC features from CT images provided by the TCIA dataset. The classifier receives the features extracted from the feature-extracting models as input and then passes them to the classifier model.

### 3.1. CT Images Dataset

The TCIA Public Access dataset was used. In this dataset, 82 abdominal contrast-enhanced 3D CT images were obtained in portal-venous from 53 male and 27 female patients 70 s after intravenous contrast injection. The subjects’ ages ranged from 18 to 76, with a mean age of 46.8 ± 16.7. The CT scans, collected using Philips and Siemens MDCT scanners at 120 kVp tube voltage, have resolutions of 512 × 512 pixels, with varied pixel sizes and slice thicknesses ranging from 1.5 to 2.5 mm [33].

### 3.2. Feature Extractors (FE)

To find common features, descriptors are compared between the PDAC CT images. For two images, we might obtain a collection of pairings with the notation (Xi,Yi)⟷$\left(Xi^{\prime },Yi^{\prime }\right)$, where (Xi,Yi) denotes a feature in one image and $\left(Xi^{\prime },Yi^{\prime }\right)$ denotes its corresponding feature in the other. There are two key techniques for FE: human feature extraction and automatic feature extraction. Machine learning models, such as SVM and decision trees, are employed for manual feature extraction [34]. However, these methods are time-consuming and inefficient for classifying PDAC CT images. This feature extraction may be carried out automatically using the deep learning technique, which is why deep learning has become so popular recently. For a given outcome, different trained and untrained models are available. In this study, three DL models, VGG16, LGBM and Inception V3, were experimented on to find the one with high accuracy when implemented as FE for the given data. Traditional FE techniques include the following:

- Statistic Pixel-Level Features (SPLF) [35]: The pixels within a segmented region can be quantitatively described by these properties. The SPL features are as follows: mean, variance, a histogram of the grey values of the pixels in the region as well as the region’s area, details on the contrast of the pixels inside the region, and edge gradients of the pixels defining the region’s boundaries;
- Feature Shape Circularity: compactness, moments, chain codes, and the Hough transform are among the characteristics that reveal details about the shape of the region boundary [36]. To describe shapes, morphological processing techniques have also been employed;
- Texture characteristics [37]: determined using the second-order statistical histogram or co-occurrence matrices, these provide information on the local texture within the region or related area of the image. Additionally, wavelet processing describes local texture information in spatial frequency analysis;
- Relational characteristics [37]: these reveal the relational and hierarchical organisation of the regions connected to a single object or a collection of objects.

This conventional method presents a challenge in that it necessitates selecting which elements in each image are crucial. Feature extraction becomes more difficult as there are more classes to categorise [38]. The CV engineer must determine which features best define various types of objects through judgement and a protracted process of trial and error. Additionally, the CV engineer must fine-tune many parameters for each feature definition.

Traditional approaches primarily rely on shape, colour, and/or texture features and their combinations; most are problem-specific and have been demonstrated to be complementary in medical images [39]. This results in a system that cannot represent high-level problem domain concepts and has poor model generalisation ability. A comprehensive model that can generate final classification labels from the raw pixels of medical images can be built using recent DL techniques.

Deep learning rejects the conventional programming paradigm, substituting issue analysis for a training framework in which the system is fed many training patterns, which are collections of inputs for which the intended outputs are known, which it learns and utilises to compute new patterns. DL is utilised in digital image processing to handle challenging issues (such as image colourisation, classification, segmentation, and detection). With massive data and plenty of processing power, DL techniques such as Convolutional Neural Networks (CNNs) have pushed the envelope of what is feasible, primarily increasing prediction performance. Superhuman accuracy is used to tackle issues previously thought to be unsolvable. This is best illustrated via image classification. In recent years, the CV field has seen significant change, largely due to the invention of CNNs, which has significantly improved object recognition [40]. A rise in computer power and the amount of data accessible for training neural networks have contributed to this recent surge in productivity.

The performance and cost-effectiveness of vision-based applications have been enhanced, further accelerating their adoption. This is due to the rapid advancements in DL and device capabilities, such as CPU power, memory capacity, power consumption, and optics [27]. Thanks to DL approaches, CV engineers may now more accurately complete tasks such as object detection, Simultaneous Localisation and Mapping (SLAM), semantic segmentation, image classification, and more. Since DL neural networks are trained rather than coded, applications utilising this method frequently require less specialised analysis and fine-tuning and take advantage of the vast amounts of video data already available in systems [27]. Furthermore, DL is more adaptable than CV algorithms, which are typically more domain-specific, because CNN models and frameworks can be retrained using a customised dataset for any use case.

**Figure 3:**
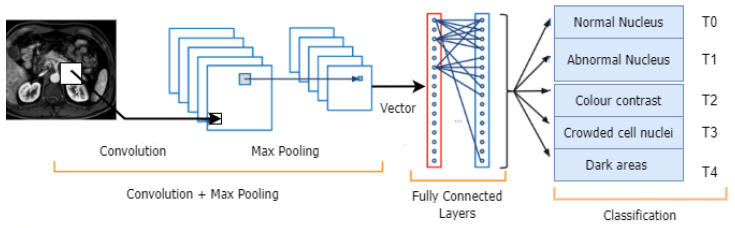
Convolutional Neural Networks’ architecture blocks.

To identify characteristics (such as edges) throughout an image, CNNs use kernels, commonly referred to as filters. A kernel is a matrix of weights trained to identify particular features. The primary principle of CNNs, Figure 3, is to spatially convolve the kernel on an input image and check for the presence of the feature it is supposed to detect, as the name suggests [41]. By computing the dot product of the kernel and the input area where the kernel is overlapped (the region of the original image the kernel is looking at is known as the receptive field), a convolution operation is performed to provide a value representing how confident it is that a specific feature is present.

The convolutional layer is frequently followed by a pooling layer to eliminate redundancy in the input feature, which speeds up the training process and lowers the amount of memory the network needs [42]. For instance, max pooling outputs the maximum value from a window moved over the input, decreasing the number of pixels output to just the crucial ones in an image. Figure 3 illustrates how deep CNNs may contain many pairs of convolutional and pooling layers. The output layer then computes the scores (confidence or probability) for the output classes/features using a dense network after a Fully Connected layer flattens the volume of the previous layer into a feature vector. After that, this output is fed into a regression function, e.g., Softmax, which converts everything into a vector whose elements add up to one.

The output of the convolution layer is added to a bias term and sent to a nonlinear activation function to speed up the learning of kernel weights [43]. Most of the time, activation functions are non-linear, including Sigmoid, TanH, and ReLU (Rectified Linear Unit). These activation functions are chosen following the characteristics of data and classification tasks. Since neurons in the brain either fire or do not, ReLUs are known to have a greater biological representation [44]. As a result, they provide sparser, more effective representations and produce positive outcomes for image identification tasks, since they are less sensitive to the vanishing gradient problem.

#### 3.2.1. LGBM Feature Extractor

LGBM is a gradient-boosting framework built on decision trees to enhance model performance while utilising less memory. The Gradient Boosting Decision Tree (GBDT) [45] framework overcomes the limitations of the histogram-based approach, which is widely used in all GBDT frameworks, by using Gradient-based One Side Sampling and Exclusive Feature Bundling (EFB) [46,47].

**Figure 4:**
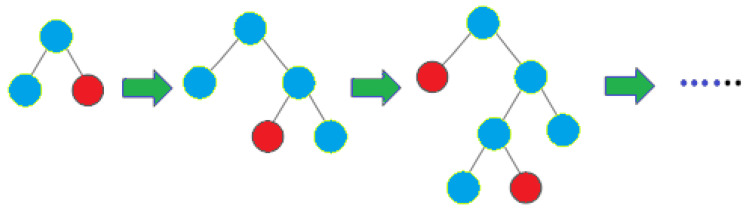
Leaf-wise tree growth of LGBM.

LGBM [48] divides the tree leaf-wise instead of growing trees level-by-level as other boosting algorithms do. The leaf chosen for growth is the one with the highest delta loss. Since the leaf is fixed, the leaf-wise algorithm has a lower loss than the level-wise algorithm. The leaf-wise tree growth could cause the model’s complexity to increase, which could potentially lead to overfitting in small samples. The diagrammatic representation of leaf-wise tree growth is shown below in Figure 4.

#### 3.2.2. VGG16 Feature Extractor

**Figure 5:**
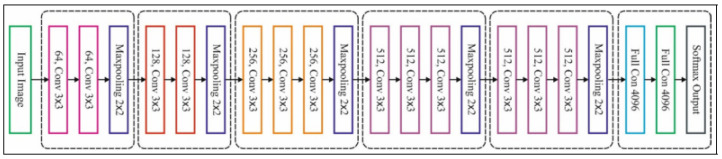
VGG16 architecture.

VGG16 (Figure 5) is a highly deep CNN for large-scale image identification [49]. The model attains 92.7% top-5 test accuracy.

##### Convolution Layer

It contains a filter that convolves over the input volume’s width and height. The output of the convolution layer is produced by performing a dot product operation between each area of the image and the filter weight content [50].

##### Pooling Layer

When drawing a pooling layer, to decrease dimensionality and complexity, speed up processing, and avoid overfitting, it uses non-linear down-sampling on the activation map [51].

##### Max Pooling

Similar to the convolutional layer, this layer has the same fundamental properties, i.e., using the maximum neighbouring value from each point in the input image is crucial. It is carried out on every individual input channel [50].

##### Softmax Layer

Using the Softmax layer, the output layer of CNN is utilised to represent the categorical distribution over labels and delivers the probability of the inputs to the labels [52].

#### 3.2.3. Inception V3

**Figure 6:**
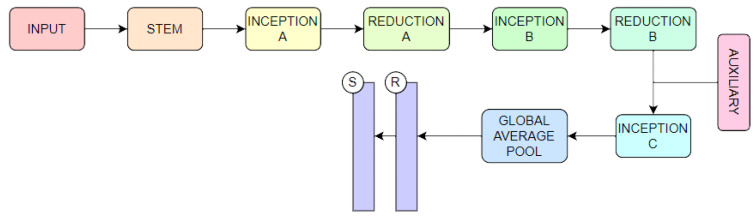
Inception V3 architecture.

Google suggested the GoogLeNet network, which is a CNN, in 2014 [53]. Inception v1 (2014), Inception v2 (2015), Inception v3 (2015) in Figure 6, Inception v4 (2016), and Inception-ResNet (2016) are the five core versions of GoogLeNet. The fundamental component of Inception v3 is a Keras-created network structure that has already been trained on Image Net. Unlike the Inception v1 and v2 network structures, the Inception v3 network structure uses a convolution kernel splitting technique [54] to separate large volume integrals into smaller convolutions.

### 3.3. Classifiers

A classifier [55] is an algorithm used in machine learning that automatically sorts data into one or more categories. In the case of this study, the classifier scans the pancreas CT images and filters them according to the class label.

#### 3.3.1. SVM Classifier

Vladimir Vapnik and his colleagues initially proposed SVM. This method of classifying aims to divide the categories along a surface that maximise the margin. The data points near the decision surface are called “support vectors”. The chosen boundary is referred to as a “hyperplane”. Using training data with a known class label, SVM [56] creates the model. Support vectors, which are essential training data elements, are created during the SVM learning stage. The model and support vectors eventually categorise the test data. SVM-based categorisation for linear data is modelled mathematically using optimisation problems [57]. The non-linear SVM idea is implemented using kernels. For the classification of non-linear data, SVM transforms the basic training data into a higher dimension via non-linear mapping. In the upper dimension, it searches for the linear separating hyperplane.

#### 3.3.2. RF Classifier

To construct a random forest, which joins a group of “weak learners” to form a “strong learner”, the Bagging approach and random variable selection were used in this study. In this technique, each decision tree in the group is constructed using a sample with replacement from the training data. Each decision tree in the group acts as a base estimator to estimate the class label of an unlabeled instance. A majority vote completes the process. For each tree in RF, Bootstrap sampling is applied, and data are separated according to the presented problem type. The Gini index is used to divide the data for classification, and each tree model can be trained to minimise the sum of squares of mean deviations for regression. The utilisation of ensembles of trees without pruning is one of the advantages of employing RFs [58].

Additionally, RF is highly resistant to overfitting and does not require standardisation or normalisation because it is unaffected by the value range. The number of trees and the features randomly sampled at each split are two factors that should be changed for the RF model. Various decision tree architectures comprise the RF [59] classifier. The individual samples from the trees connected to this classifier are chosen randomly.

#### 3.3.3. XGBoost Classifier

**Figure 7:**
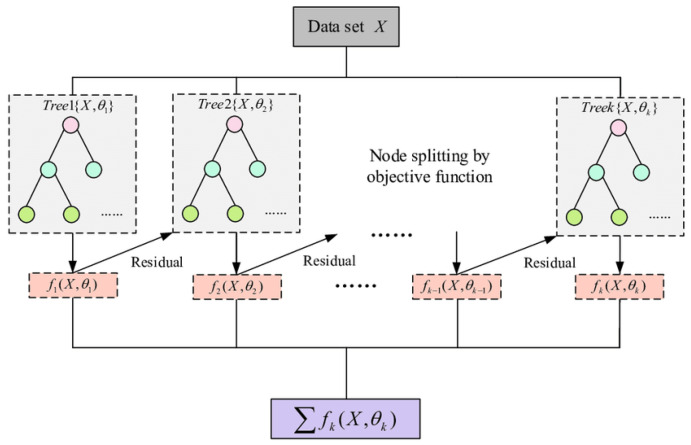
XGBoost architecture.

Training is sped up and overfitting is decreased using XGBoost [60] randomisation techniques such as random subsamples and column subsampling. XGBoost decreases the cost of computing the ideal split by storing the data in a compressed, pre-sorted column-based system. This column-based storage structure enables a parallel search for the optimal division of each taken-into-consideration attribute. Additionally, rather than scanning all potential candidate splits, the XGBoost uses a data percentiles-based strategy to assess a smaller subset of candidate splits and calculate their gain using combined statistics. Therefore, the node-level data subsampling has come close to realising this concept. XGBoost uses a sparsity-aware approach to exclude null values from the split candidate’s loss gain calculation. The XGBoost architecture is demonstrated in Figure 7.

### 3.4. Proposed Model

**Figure 8:**
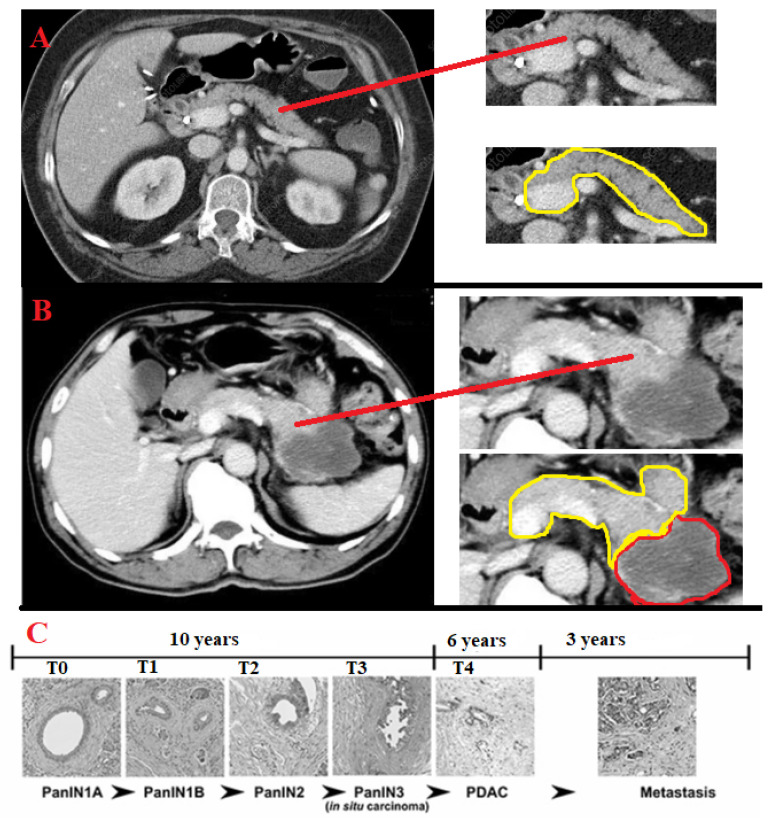
(**A**) A normal pancreas CT image; (**B**) PDAC at an advanced stage, with spreading to other organs; (**C**) Stages of PDAC development, which are represented as T0 to T4. The other stage, metastasis, is not represented because cancer at that point has spread to other organs surrounding the pancreas. PanIN (pancreatic intraepithelial neoplasia) with low-grade pancreatic intraepithelial neoplasia encompasses three older terms—PanIN-1A, PanIN-1B, and PanIN-2. These include Pancreatic Intraepithelial Neoplasia 1-A, Pancreatic Intraepithelial Neoplasia 1-B, and Pancreatic Intraepithelial Neoplasia 2. Pancreatic Intraepithelial Neoplasia 3 is high-grade pancreatic intraepithelial neoplasia, which then advances to PDAC.

PDAC arises from macroscopic cystic or microscopic precursor lesions [61]. Through the accumulation of genetic and epigenetic alterations, the normal ductal epithelium can progress to a series of precursor lesions, invasive carcinoma, and, eventually, metastatic carcinoma, as shown in Figure 8.

**Figure 9:**
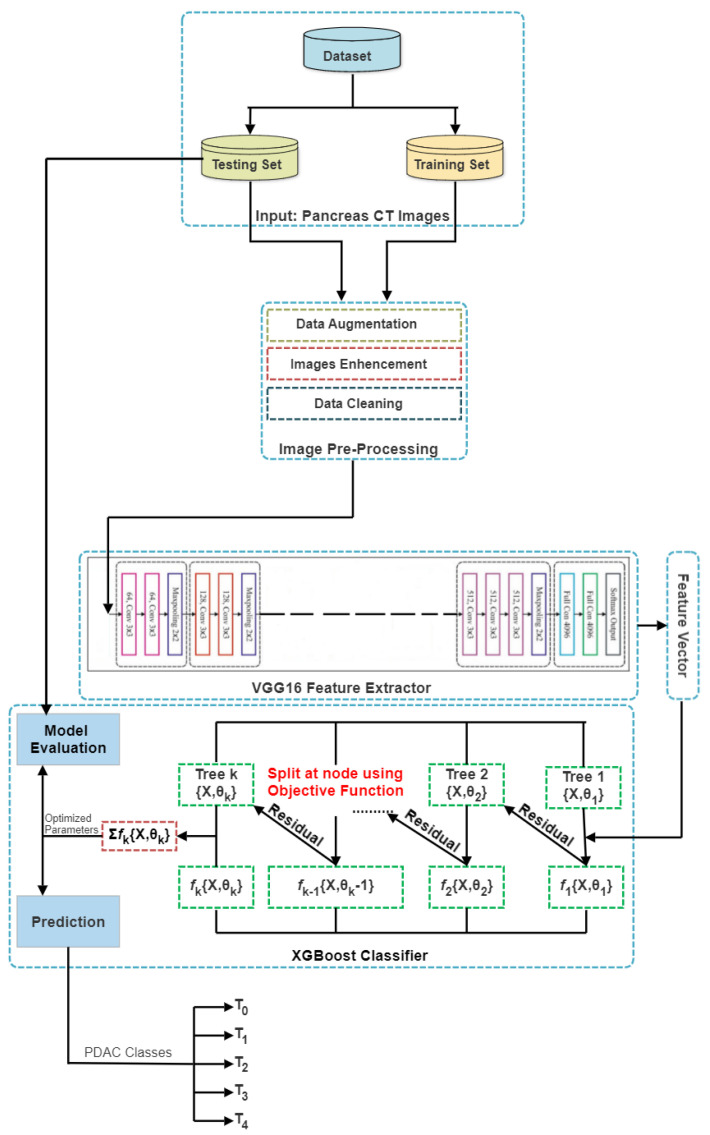
Proposed model, illustrating VGG16 as the feature extractor and XGBoost as the classifier.

This study suggests a hybrid VGG16–XGBoost model (Figure 9) to sort pancreas CT images into T0, T1, T2, T3, and T4 classes; for this task, we use an XGBoost classifier. A technique for machine learning that performs classification and regression modelling is called XGBoost. This system consists of a set of gradient-boosted decision trees. New models are created using the gradient boosting technique to anticipate the errors or residuals of prior models, which are then integrated to obtain the final prediction. To reduce errors, boosting algorithms employ gradient descent techniques.

## 4. Experiments

Data were split, and the training set was composed of 80% of the data, while the test set was 20%, i.e., 229 images used for training and 49 images used for testing. The classification job was trained for RF, SVM, and XGBoost. The proposed multi-feature combination model exhibited the greatest performance when the following performance metrics were utilised to assess the proposed system: accuracy (ACC), precision (Pre), recall (Rec), and F1-score (F1). The XGBoost classifier outperformed the RF and SVM classification algorithms regarding performance (Algorithms 1 and 2).

We also adjusted XGBoost hyperparameters to test which settings produced better results. The following parameters were then used: model_colsample_bytree = 0.9, model_gamma = 0.0, model_learning_rate = 0.01, model_max_depth = 5, model_min_child_weight = 1, model_n_estimators = 250, model_reg_lambda = 1, and model_subsample = 0.5

Keras, an open-source Python interface for Convolutional Neural Networks, was used as the library. Keras is a TensorFlow library interface. One Hot Encoding was performed to encode categorical features for machine learning models. This type of encoding creates a new binary feature for each viable category and assigns a value of 1 to each sample’s feature that corresponds to its original category. The Jupyter Notebook is a tremendously effective tool for generating and presenting deep learning projects interactively.

Ductal adenocarcinomas typically present as poorly defined masses surrounded by the broad desmoplastic response. They enhance weakly compared to normal pancreatic tissue and seem hypoattenuating on arterial phase scans in 75–90% cases. However, they may become iso-attenuating on delayed scans, necessitating multiple-phase scanning when pancreatic cancer is suspected. The double duct indicator is visible. Calcifications in adenocarcinoma are exceedingly rare and are more likely to result from a pre-existing condition, such as chronic pancreatitis [62].
**Algorithm 1:** Model algorithm: Implementing LGBM to Classifiers**Input**: Input: train_images.**Input**: Input: test_labels. 1/* Read training and testing data.*/ 2Read input images and assign labels based on folder names. 3Resize images. 4Capture images and labels into arrays. /* Start by creating empty lists. Do the same for test/validation images. */ 5Encode labels. /* from the text (folder names) to integers. */ 6Separate the data into train and test datasets. /* Already split but assigned to a meaningful convention. Split the dataset here if there is just one. */ 7Normalise pixel values to between 0 and 1. 8Feature extractor function. /* Input shape is (n, x, y, c) - number of images, x, y, and channels */ 9Create a temporary data frame. /* To capture information for each loop. Reset the data frame to blank after each loop. */10Start adding data to the data frame.11Add features from the current image to the item in the dataset.12Extract features from training images.13Reshape to a vector. /* For Random Forest/SVM training */14Define the classifier.15Model Train.16/* Using the training data. */17Send test data using the identical feature extractor technique.18Make prediction. /* Use the learned RF model. */19Extract features. /* Also reshape them to the right dimensions */20Predict.21**return** Classification_Report

Regarding predicting unresectability, CT correlates strongly with surgical results, with a positive predictive value of 89–100%. The most significant aspect of evaluating locally is the tumour’s connection to nearby arteries, including the superior mesenteric artery and the celiac axis. T4 illness is unresectable if a tumour covers a vessel by more than 180 degrees [63].   
**Algorithm 2:** Model algorithm: Implementing VGG16 to Classifiers**Input**: Input: train_images.**Input**: Input: test_labels. 1/* Read training and testing data. */ 2Read input images and assign labels. /* Based on folder names */ 3Resize images. 4Capture images and labels into arrays. /* Start by creating empty lists. Do the same for test/validation images. */ 5Encode labels. /* From the text (folder names) to integers. */ 6Separate the data into train and test datasets. /* Already split but assigned to a meaningful convention. Split the dataset here if there is just one. */ 7Normalise pixel values to between 0 and 1. 8One hot encode y values. /* For the neural network. */ 9Load model. /* Without classifier/fully connected layers */10Make loaded layers non-trainable. /* It is important to work with pre-trained weights. */11/* Now, let us use features from the convolutional network */12Define the classifier.13Train the model. /* Using the training data. */14Send test data. /* Using the identical feature extractor technique. */15Make a prediction. /* Use the learned RF model. */16Extract features and reshape them to the right dimensions.17Predict.18**return** Classification_Report

## 5. Experimental Results

The XGBoost classifier outperformed the RF and SVM classification algorithms regarding performance. As a result, the ACC’s correct recognition rate when XGBoost was utilised as a classifier was 97%. These findings suggest that the suggested strategy might aid in a more precise diagnosis of instances that are challenging to categorise in pancreas CT imaging.

**Figure 10:**
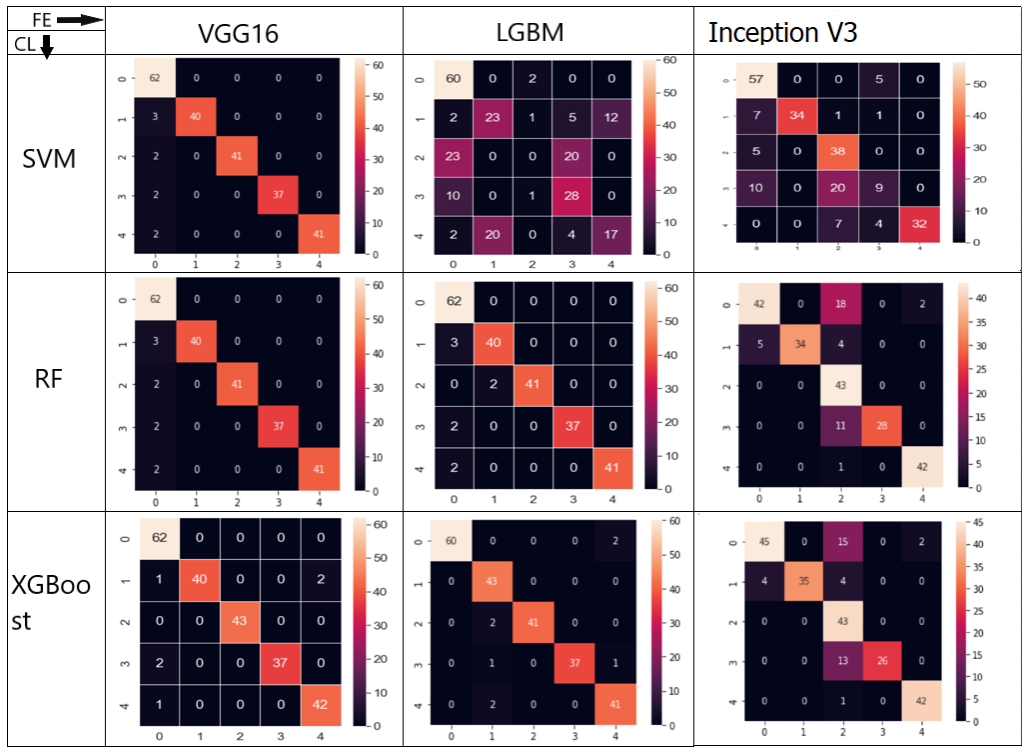
Confusion Matrix for using VGG16–XGBoost, LGBM–XGBoost, Inception V3–XGBoost, VGG16–RF, LGBM–RF, Inception V3–RF, VGG16–SVM, LGBM–SVM, and Inception V3–SVM combinations. The different classification (CL) models with the used feature extraction (FE) models are shown.

Performance Assessment Transfer learning was used to assess the effectiveness of the pre-trained VGG-16 model using the precision, recall, and F1 score metrics. In the equations, Equations (1)–(5), the words True Positive (TP), False Negative (FN), True Negative (TN), and False Positive (FP) are employed. A confusion matrix is a table-based illustration of the effectiveness of the prediction model. Each entry in a confusion matrix indicates the number of predictions the model made when correctly or incorrectly categorising the classes (Figure 10).

Utilising a confusion matrix as the deep learning model’s evaluation criterion is usually preferable. It provides very straightforward yet effective performance metrics for the model. The confusion matrix’s most widely used performance metrics are as follows:

(1) $$Accuracy=\frac{TP+TN}{TP+TN+FP+FN}$$

(2) $$Misclassification\;Rate=\frac{FP+FN}{TP+TN+FP+FN}$$

(3) $$Precision=\frac{TP}{TP+FP}$$

(4) $$Recall=\frac{TP}{TP+FN}$$

(5) $$F1\text{-}score=\frac{2TP}{2TP+FP+FN}$$

In an ideal case, we would like a model with a precision of 1 and a recall of 1, which amounts to an F1 score of 1 or 100% accuracy, which is typically not the case for a machine learning model. We must attempt to increase precision while maintaining a greater recall value. A confusion matrix can assess the model’s recall, precision, and accuracy performance.

Table 1, Table 2 and Table 3 show the performance measures for using LGBM, VGG16, and Inception V3 as the backbone implemented for feature extraction to XGBoost, RF, and SVM as the classifiers. XGBoost using VGG16 had the highest ACC (Accuracy), WP (Weighted Precision), and WFS (Weighted F1-Score) as performance measures.

### ROC

**Figure 11:**
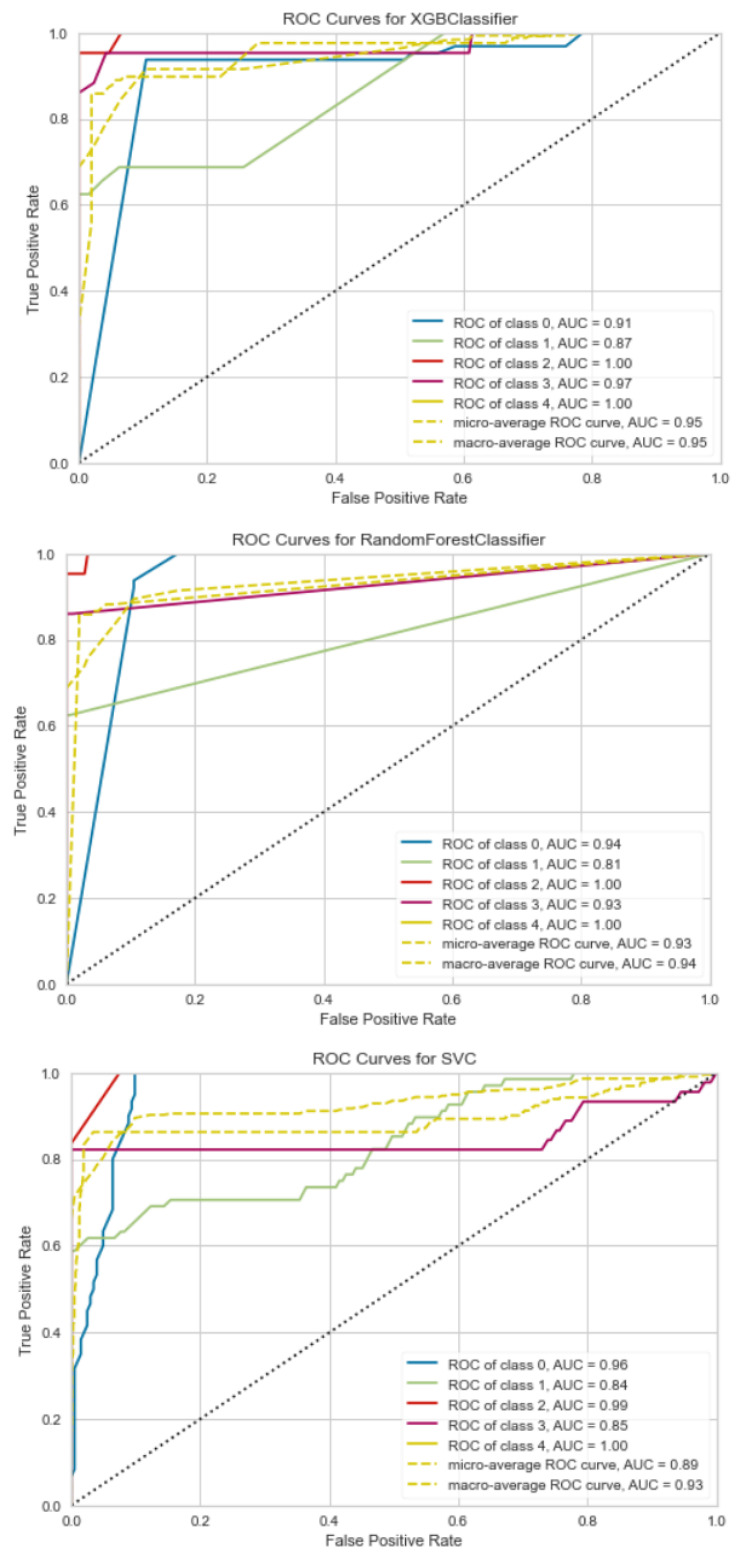
ROC curves for SVM, RF, and XGBoost, showing True Positive against False Positive rates.

A graph depicting the effectiveness of a classification model at all classification thresholds is called a ROC curve (receiver operating characteristic curve) (Figure 11). The True Positive and False Positive rates are plotted on this curve. The chart demonstrates the connection between the True and False Positive rates. It was decided to compare each class to every other using the One-vs.-Rest methodology. The interpretation of the AUC score os as follows:

1. ≤0.5 = There is no prejudice;
2. 0.5–0.7 = Inadequate discrimination;
3. 0.7–0.8 = Acceptable discrimination;
4. 0.8–0.9 = Good discrimination;
5. 0.9 = Excellent discrimination.

Although all the models produce acceptable AUC scores, XGBoost had the highest score, followed by Random Forest, and then SVM. The higher the AUC, the better the model predicts 0 classes as 0 and 1 classes as 1. By analogy, the higher the AUC, the better the model distinguishes between images with and without PDAC.

The model helps to diagnose the stages of progression for PDAC tumour cells. Surgery for stages T0 and T1 offers a potential cure but comes with a significant morbidity of 20–30% and a fatality rate of 5%. A Whipple procedure is used to remove pancreatic head tumours [63]. Even when resection is possible, most patients die from recurrence, barely doubling survival in operated patients after five years, from 5% to 10%. Almost a fifth of individuals will have died 12 months after their diagnosis. This makes it necessary to have models of high accuracy to diagnose PDAC, and the VGG16–XGBoost hybrid model with a classification accuracy of 0.97 will be a good tool in medical imaging [64].

## 6. Discussion

As shown in Figure 10, combining VGG16 and XGBoost results in a lower misclassification rate than other models, indicating that the model can accurately identify place-infected regions. Our approach can accurately identify those positive locations, even if the input patch only has a minor PDAC tumour presence. These findings suggest that our model recognised PDAC mostly through nuclear characteristics. Our system identified tumour cell nuclei as large, irregular, crowded, and dark regions. For tumour identification, these characteristics are essential. However, there are also False Positive and False Negative locations, including those overpopulated with nuclei.

Additionally, we found that the classification model tended to identify packed and asymmetrical groupings of aberrant cell nuclei, wherein the model may be making judgements based on colour contrast. The distribution of cell nuclei is asymmetrical in a few False Positive locations.

Low accuracy is obtained when using LGBM as the feature extractor. The SVM classifier had 0.56 accuracy, while RF and XGBoost had 0.92 and 0.94, respectively. Using VGG16 as the feature extractor resulted in all the classifiers producing the highest accuracy, compared to Inception V3 and SVM. For Inception V3, the classifiers had an accuracy of 0.63 (SVM), 0.86 (RF), and 0.95 (XGBoost), while for VGG16, the classifiers’ accuracy was 0.95 (SVM), 0.96 (RF), and 0.98 (XGBoost). Combining VGG16 and XGBoost models resulted in better accuracy than single end-to-end models, as shown in Table 4.

Shape characteristics, colour histogram features, colour moment features, and texture features are the most often utilised features in the classification of medical images, according to [67]. Most of the earlier studies use global characteristics to categorise medical images. The most often utilised attributes for recognising targets are texture and colour moment, which are both global features. In [68], an SVM classifier produced SE = 93 and SP = 92 after they retrieved colour, texture, and form variables. To perform well on a dataset of 217 melanomas and 588 photos, [69,70] used colour moment characteristics and texture features.

The Twin SVM model had good results of 0.98, contributing to implementing combined models to form a hybrid. To address the CV issues mentioned above, classic machine learning classification algorithms such as Support Vector Machines and K-Nearest Neighbours are typically supplemented with feature descriptors such as Scale Invariant Feature Transform (SIFT) and Speeded Up Robust Features (SURF) [71]. These days, classical techniques are utilised when the problem can be simplified so that it can be implemented on low-cost microcontrollers or to restrict the problem for deep learning techniques by emphasising specific elements in the data, enhancing the data, or helping with dataset annotation.

To classify breast cancer into carcinoma and noncarcinoma using histopathology images, [72] presented an ensemble DL technique. In this instance, the framework was created using the VGG16 and VGG19 models. Three further convolutional layers are added to the same fundamental design of VGG16 to create VGG19. The models were adjusted during training, except for the first block, to improve performance. An overall accuracy 95.29% was obtained after the combined tuned VGG16 and VGG19 models.

Algorithms for machine learning have their limits, and it might not be easy to create a model with a high degree of accuracy. The accuracy of 0.98, which is a significant improvement over past research, shows that, if we create and combine many models, we have the potential to increase the overall accuracy. Thus, greater resilience is a fundamental benefit of using the VGG16–XGBoost model. For a specific dataset, one method might not be able to predict the outcome perfectly. By dividing the FE and classification tasks to be completed by separate models, the model reduces the burden on one model once more. As a result, the task division does not put demand on one algorithm’s resources.

## 7. Conclusions

Deep learning techniques are viable in real-world situations, and the structures benefit from the learning process. They have been used in relation to medical imaging and this application might develop quickly in the upcoming years. Deep learning in medical imaging has significant implications for the treatment. Significantly, this field of study guarantees that patients will receive better care. The advantages of deep learning approaches are crucial to demonstrating that they are used most effectively. Deep learning algorithms can categorise, classify, and count illness patterns from image processing in medical image analysis. Additionally, they enable the expansion of analytical objectives and produce therapy prediction models for patients. These difficulties are being taken into account by researchers in medical imaging, and deep learning is blossoming in this field.

Similar to deep learning in many other applications other than health care, it is advancing quickly. A fast-developing area of clinical image analysis called “radiomics” has great potential to help with cancer diagnosis and treatment decision-making. To implement modern computer vision and machine learning techniques effectively, this goal of more effective decision-making requires significant individual and combined expertise from domain specialists in medicine, biology, and computer science. If the raw data are made available, deep learning and machine learning can considerably progress the field of radiomics in the upcoming years, regardless of the patient population or type of tumour.

One of the limitations of this research is that, due to limited datasets for PDAC, we were limited to testing the model on one dataset. This does not limit the implementation of the model to another dataset. For future work, we will experiment on other datasets and try to use the ensemble method for feature extraction so that some models may detect hidden features.

To catch up to other imaging fields, deep models’ applications in the medical image analysis domain need a lot of work to be put in because their huge dataset requirements for excellent feature extraction make them inefficient for small datasets. However, medical datasets are frequently tiny because medical images are frequently challenging. As a result, if we employ a deep model to address a limited dataset directly, the method will likely result in model overfitting. Aside from these issues, the model’s interpretability is fairly poor, and training a deep model often involves significant computing.

## Figures and Tables

**Table 1:** Evaluation measures results for the model using LGBM (Light Gradient Boosting Machine) as a feature extractor and experiments carried out on three classifiers (CF), that is, SVM (Support Vector Machines), RF (Random Forest), and XGBoost (Extreme Gradient Boosting). The metrics used are AV (Averages), that is, MAV (Micro Average) and WAV (Weighted Average), ACC (Accuracy), WP (Weighted Precision), WFS (Weighted F1 Score), PR (Precision), RE (Recall), and FS (F10Score).

CF	ACC	WP	WFS	CL	AV	PR	RE	FS
SVM	0.56	0.46	0.49					
					MAV	0.45	0.52	0.47
					WAV	0.46	0.56	0.49
				T0		0.62	0.97	0.75
				T1		0.53	0.53	0.53
				T2		0.57	0.60	0.57
				T3		0.49	0.72	0.58
				T4		0.59	0.40	0.47
RF	0.92	0.91	0.93					
					MAV	0.97	0.96	0.96
					WAV	0.96	0.96	0.96
				T0		0.90	1.00	0.95
				T1		0.95	0.93	0.94
				T2		0.97	0.95	0.98
				T3		0.97	0.95	0.97
				T4		0.97	0.95	0.98
XGBoost	0.94	0.93	0.93					
					MAV	0.97	0.96	0.96
					WAV	0.96	0.96	0.96
				T0		0.90	1.00	0.95
				T1		0.95	0.93	0.94
				T2		1.0	0.95	0.98
				T3		0.95	0.95	0.97
				T4		0.94	0.95	0.97

**Table 2:** Evaluation measures results for the model using VGG16 (a Convolutional Neural Network of 16 convolutional layers by Visual Geometry Group) as a feature extractor and experiments carried out on three classifiers (CF), that is, SVM (Support Vector Machines), RF (Random Forest), and XGBoost (Extreme Gradient Boosting). The metrics used are AV (Averages), that is, MAV (Micro Average) and WAV (Weighted Average), ACC (Accuracy), WP (Weighted Precision), WFS (Weighted F1 Score), PR (Precision), RE (Recall), and FS (F1-Score).

CF	ACC	WP	WFS	CL	AV	PR	RE	FS
SVM	0.95	0.96	0.94					
					MAV	0.97	0.96	0.96
					WAV	0.97	0.96	0.96
				T0		0.87	1.00	0.93
				T1		1.00	0.93	0.96
				T2		1.00	0.95	0.98
				T3		1.00	0.95	0.97
				T4		1.00	0.95	0.96
RF	0.96	0.95	0.95					
					MAV	0.97	0.96	0.96
					WAV	0.97	0.96	0.96
				T0		0.87	1.00	0.93
				T1		1.00	0.93	0.96
				T2		1.00	0.95	0.98
				T3		1.00	0.95	0.97
				T4		1.00	0.95	0.98
XGBoost	0.98	0.98	0.97					
					MAV	0.98	0.97	0.97
					WAV	0.98	0.97	0.97
				T0		0.94	1.00	0.97
				T1		1.00	0.94	0.96
				T2		1.0	1.00	1.00
				T3		1.00	0.95	0.98
				T4		0.96	0.98	0.99

**Table 3:** Evaluation measures results for the model using Inception V3 as a feature extractor and experiments carried out on three classifiers (CF), that is, SVM (Support Vector Machines), RF (Random Forest), and XGBoost (Extreme Gradient Boosting). The metrics used are AV (Averages), that is, MAV (Micro Average) and WAV (Weighted Average), ACC (Accuracy), WP (Weighted Precision), WFS (Weighted F1 Score), PR (Precision), RE (Recall), and FS (F1-Score).

CF	ACC	WP	WFS	CL	AV	PR	RE	FS
SVM	0.63	0.60	0.61					
					MAV	0.57	0.62	0.58
					WAV	0.55	0.60	0.58
				T0		0.68	0.94	0.62
				T1		0.58	0.62	0.60
				T2		0.59	0.59	0.67
				T3		0.60	0.69	0.61
				T4		0.68	0.68	0.51
RF	0.86	0.87	0.89					
					MAV	0.89	0.90	0.92
					WAV	0.87	0.91	0.90
				T0		0.90	0.90	0.92
				T1		0.91	0.89	0.89
				T2		0.89	0.89	0.89
				T3		0.89	0.88	0.91
				T4		0.87	0.91	0.91
XGBoost	0.95	0.95	0.95					
					MAV	0.92	0.93	0.94
					WAV	0.91	0.91	0.93
				T0		0.90	0.96	0.93
				T1		0.91	0.95	0.95
				T2		0.92	0.95	0.95
				T3		0.93	0.95	0.94
				T4		0.93	0.94	0.95

**Table 4:** Comparison table for accuracy obtained when applying different models in diagnosis.

Paper	Application	Model	Accuracy
[23]	Diabetes Prediction	K-Nearest Neighbour, SVM, Random Forest, LGBM, AdaBoost, and Decision Tree	89.85%
[24]	Identify Colon Cancer	SVM	94.51%
[25]	Identify COVID-19	Random Forest + AdaBoost	94%
[65]	Pancreatic Cystic Lesion	CNN of VGG16	93.11%
[66]	Pancreatic Cancer	Twin SVM	98%
[67]	Skin Cancer		
		CNN	83.2%
		ResNet50	83.7%
		Inception V3	85.8%
		Inception ResNet	84%

## Data Availability

A publicly available benchmarked dataset was used in this research.
